# Widespread methane seepage along the continental margin off Svalbard - from Bjørnøya to Kongsfjorden

**DOI:** 10.1038/srep42997

**Published:** 2017-02-23

**Authors:** S. Mau, M. Römer, M. E. Torres, I. Bussmann, T. Pape, E. Damm, P. Geprägs, P. Wintersteller, C.-W. Hsu, M. Loher, G. Bohrmann

**Affiliations:** 1 MARUM – Center for Marine Environmental Sciences and Department of Geosciences, University of Bremen, Klagenfurter Str., 28359 Bremen, Germany; 2 College of Oceanic and Atmospheric Sciences, Oregon State University, 104 Ocean Admin Building, Corvallis, Oregon 97331–5503, USA; 3 Alfred Wegener Institute Helmholtz Centre for Polar and Marine Research, Am Handelshafen 12, 27570 Bremerhaven, Germany

## Abstract

Numerous articles have recently reported on gas seepage offshore Svalbard, because the gas emission from these Arctic sediments was thought to result from gas hydrate dissociation, possibly triggered by anthropogenic ocean warming. We report on findings of a much broader seepage area, extending from 74° to 79°, where more than a thousand gas discharge sites were imaged as acoustic flares. The gas discharge occurs in water depths at and shallower than the upper edge of the gas hydrate stability zone and generates a dissolved methane plume that is hundreds of kilometer in length. Data collected in the summer of 2015 revealed that 0.02–7.7% of the dissolved methane was aerobically oxidized by microbes and a minor fraction (0.07%) was transferred to the atmosphere during periods of low wind speeds. Most flares were detected in the vicinity of the Hornsund Fracture Zone, leading us to postulate that the gas ascends along this fracture zone. The methane discharges on bathymetric highs characterized by sonic hard grounds, whereas glaciomarine and Holocene sediments in the troughs apparently limit seepage. The large scale seepage reported here is not caused by anthropogenic warming.

Methane is, after water vapor and CO_2_, the most abundant greenhouse gas on Earth. When averaged over a 100 yr timescale, the warming effect of methane per unit mass is 28 times higher than that of CO_2_^1^. Methane is produced in oceanic sediments either by methanogens at temperatures typically below ~80 °C, or through the breakdown of organic molecules at higher temperatures^2^,^3^. Buoyancy and pressure gradients can drive gas advection to shallower sediments where methane can be consumed via anaerobic oxidation of methane (AOM)^4^ at the sulfate-methane transition zone and aerobic methane oxidation at the sediment surface^5^. Methane can also be sequestered within a cage of water molecules, in a gas hydrate structure, stable under the low temperature and high pressure conditions that define the gas hydrate stability zone^6^.

If the upward methane flux is not fully exhausted by these processes, methane is emitted to the ocean either dissolved in the venting fluids or, in case of over-saturation, as gas bubbles^7^. As the bubbles ascend through the water column, a fraction of the methane gas dissolves^8^, generating patches of high methane concentration^9^. When the gas discharge is persistent and vigorous, it leads to the formation of large dissolved methane plumes. The dissolved methane is diluted by mixing with the surrounding ocean water and it is further oxidized by aerobic methanotrophs. Only in cases where dissolved methane reaches the surface-mixed layer in concentrations above saturation, can it be transferred to the atmosphere via sea-air gas exchange^10^. At present, the oceanic methane source to the atmosphere is very small (2–10%)^11^, as it is limited to emissions from vigorous and shallow seeps (<100 m)^1^,^7^,^8^. There is, however, an ongoing controversy regarding the methane discharge from sediments during warming events throughout Earth’s history^12^,^13^, as a temperature increase could potentially destabilize gas hydrates and liberate vast quantities of methane. Such a mechanism has been proposed to explain the seepage observed offshore Svalbard^14^.

Along the Svalbard margin, changes in glacier extent, anthropogenic ocean warming, and seasonal bottom water fluctuations have all been considered as controls on gas hydrate stability. During the last glacial period, gas hydrate formation beneath the ice sheet is thought to have extended to the continental shelf edge, potentially storing large amounts of methane^15^. Post-glacial warming and the resulting release of the previously sequestered methane has been linked to formation of the numerous pockmarks in this Arctic region. Recent climate models predict that the rise in both atmospheric and oceanic temperatures in the Norwegian-Svalbard region will be greater than in other locations at a similar latitude because of the combined effects of warmer inflowing Atlantic Water^16^ and reduced sea ice cover^17^. Recent increase in bottom water temperature and the observation of gas emissions offshore Prins Karls Forland (PKF) at a water depth close to the landward limit of the gas hydrate stability (~400 m) were used to link the gas discharge to hydrate dissociation induced by anthropogenic warming^14^. More recently, Berndt *et al*.^18^ challenged this conclusion by showing that seasonal fluctuations in bottom water temperature can significantly shift the upper edge of hydrate stability to shallower or deeper water, thus providing a natural mechanism for a potential methane release from hydrates. These authors also show that the methane-derived carbonates associated with the PKF seeps are ~3000 years old, and thus the methane seepage must have been active long before the onset of anthropogenic warming.

We questioned the uniqueness of the seepage offshore PKF, since the tectonic and sedimentary characteristics of the PKF sites extend southward along and beyond Svalbard. The predominant tectonic feature of the area is the Hornsund Fracture Zone (HFZ, Fig. 1) that runs from 74° to 79°N and marks the continent-ocean boundary^19^. With the opening of the northern Atlantic in the late Eocene, extension in this zone was responsible for down-faulting of blocks on the western side of HFZ^20^ and thinning of the continental crust, which caused subsidence and accumulation of a thick sequence of Cenozoic sediments on the outer part of the continental shelf 20,21. During the ice-ages (Pliocene–Pleistocene), the continental shelf was further shaped by ice sheet propagation and retreat over the entire margin from Svalbard to northern Norway^22^,^23^. Troughs and banks, prominent bathymetric features of the shelf, are thought to have focused ice movements, so that fast flowing ice streams extended and filled the cross shelf troughs, while less dynamic ice flows deposited their sediment loads over the banks. Ridges, interpreted as moraines, are observed parallel to the coast on the shallow shelf-banks, whereas, in the troughs, till deposits are buried beneath glaciomarine and Holocene sediments^24^. In order to identify if the PKF is a special geological site or if the seepage is related to large scale tectonic structures and sedimentary deposits, we surveyed a larger area of this margin to explore seepage extent from the upper edge of gas hydrate stability (400 m) to the shelf (33 m). Furthermore, we constrained the fate of the discharged methane in the water column to evaluate the significance of the gas discharge to the global atmospheric methane budget.

## Results

### Flare locations and sub-seafloor structures

**Figure 1:**
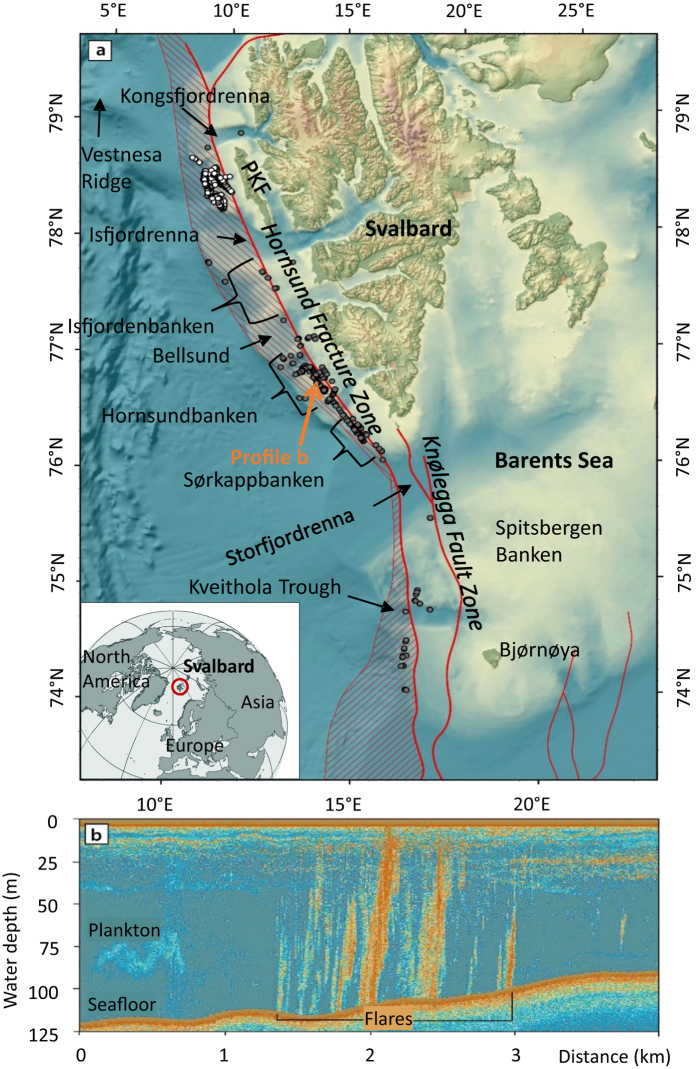
Flare locations along the Svalbard margin. (**a**) Flares observed during HE449 and HE450 (gray dots) as well as flares described by Sahling *et al*.^25^ off Prins Karls Forland (PKF) (white dots). The map shows the main structural features in red according to Faleide *et al*.^72^; the striped fields indicate the stretched continental crust (stripes from left top to right bottom) and the Tertiary volcanic province (stripes from left bottom to right top). (**b**) Flares at Hornsundbanken reach the sea-surface (orange arrow marks the location in (**a**)). The map was generated using ArcGIS 10.2 (©ESRI).

During a six week survey in the summer of 2015, we discovered over a thousand bubble emission sites from Bjørnøya to Kongsfjordrenna (Fig. 1, Supplementary Fig. S1). Bubble emissions, imaged as flares in the hydroacoustic records, were observed in water depths from 33 to 429 m with a median of 103 m. Several of the flares on the shelf (shallower than ~120 m water depth) extend from the seafloor to the sea surface (Fig. 1b). Between 74°N and 75°N, flares occur west and northwest off Bjørnøya on the Spitsbergenbanken. Flares disappeared at the edges of Kveithola Trough and Storfjordrenna. Farther north (from 76°N to 77°N), flare clusters were found along Sørkappbanken and Hornsundbanken. Less flares were observed on Isfjordbanken; a single flare was found in Bellsund; and no flares were seen in the trough of Isfjordrenna. Sahling *et al*.^25^ had previously documented the occurrence of flares on the shelf of PKF at ~78.5°N; we included their data in this study for completion (Fig. 1a). Additional flares were observed in Kongsfjordrenna, which marks the northernmost extent of our survey at 78.9°N. In general, flares were more prevalent on the shelf than on the shelf edge, where only a few isolated flares were observed. Most of the flares occurred on topographic highs, whereas only a few flares were observed in the troughs between the banks.

Acoustic seafloor penetration was low or non-existing at most of the flare sites (Supplementary Fig. S2), indicating sonic hard grounds. Most (72%) of the gas discharge sites were located on banks or regional highs: Sørkappbanken, Hornsundbanken, Isfjordenbanken, and offshore PKF; 44% of these flares were located in areas without any sub-bottom reflections and 28% were situated where slight bottom penetration revealed chaotic structures beneath the seafloor. Only a small fraction (28%) of the flares occurred in regions of low to moderate bottom-penetration with visible parallel reflections beneath the seafloor. These were situated around Bjørnøya and in Kongsfjordrenna, but a few flares at Hornsundbanken and Sørkappbanken were also found above locations that exhibit parallel seafloor reflections. Due to the low acoustic seafloor penetration, sub-bottom features such as blank zones indicative of gas conduits, could not be imaged.

### Hydrography

**Figure 2:**
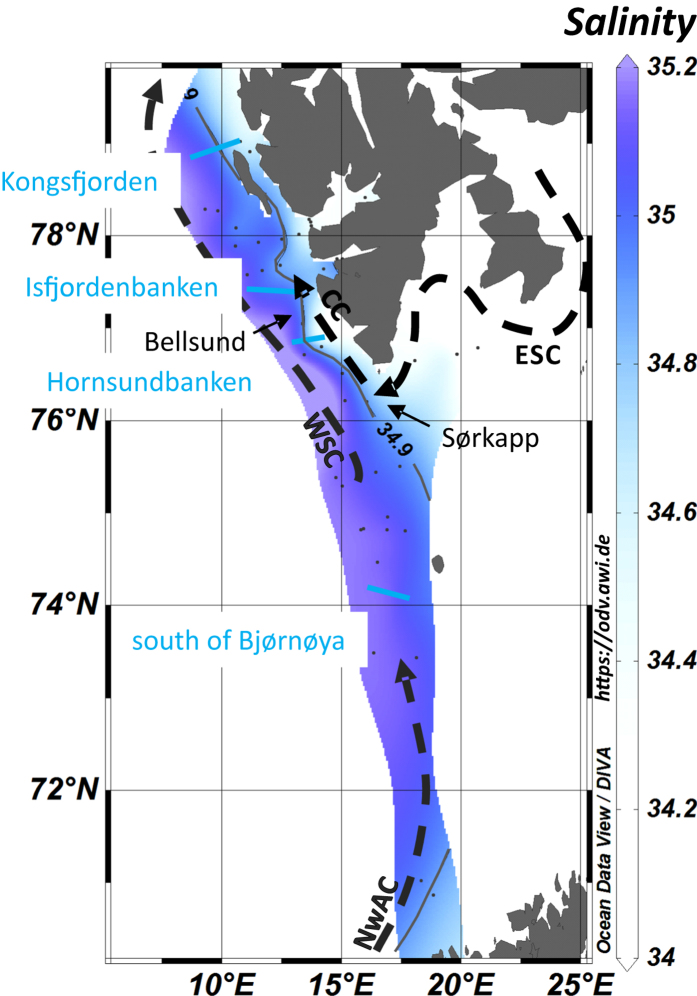
Contour plot of salinities observed in the 100–200 m water depth layer. The contour of a salinity of 34.9 indicates the Arctic front between saline, warm Atlantic Water and fresher, cold Arctic Surface Water. The Atlantic Water is carried by the Norwegian Atlantic Current (NwAC) and the West Spitsbergen Current (WSC) (dark gray arrows). The Arctic Surface Water is carried by the East Spitsbergen Current (ESC) and the Coastal Current (CC) (black arrows). The locations of the transects of Supplementary Figure S2 online are shown as blue lines. The water mass Melt/Surface Water (MSW) occurred in depths shallower than 100 m and is, thus, not shown here. The plot was generated using Ocean Data View Version 4.5.7 (https://odv.awi.de) and DIVA gridding.

Atlantic Water (AW) with salinities >34.9 and temperatures >3 °C^26^ is transported northwards by the Norwegian Atlantic Current (NwAC) and West Spitsbergen Current (WSC). AW was the dominant water mass around Bjørnøya during our surveys (Fig. 2), where saline water was overlain by less than 50 m of Arctic Surface Water (ASW) with salinities between 34.4 and 34.9^27^ (Supplementary Fig. S3). A large input of low salinity water occurred at Sørkappbanken, where the East Spitsbergen Current (ESC) carried ASW and Melt/Surface Water (MSW) with salinities <34.4^28^ onto the western shelf of Svalbard. The low saline waters continued to flow northward on the inner western shelf, in what is known as the Coastal Current (CC)^28^. ASW and MSW dominated in areas shallower than 100 m and formed a low salinity layer that extended tens of kilometers seaward.

Topography steers the AW onto the shelf and fjords, so that the AW appears to meander along the coast. The AW extends onto the shelf where troughs are located and is confined to the slope where topographic highs are situated (Fig. 2). For example, AW flowed into Bellsund (a trough), but did not reach Hornsundbanken during our surveys.

### Methane concentrations and dissolved methane inventory

**Figure 3:**
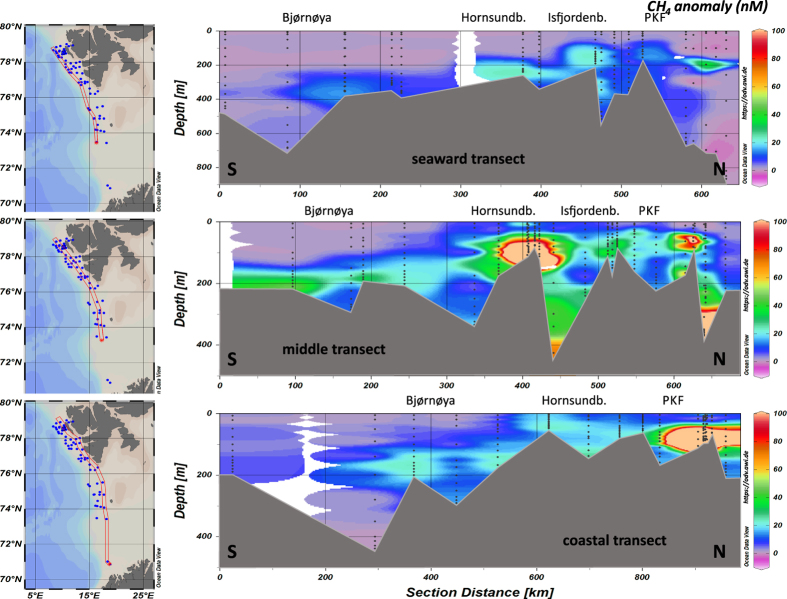
South to north transects of dissolved methane concentration anomalies. Methane anomalies were derived by subtracting the atmospheric methane equilibrium concentrations from the measured methane concentrations. The maps indicate the transect location and the red star marks the starting point. Above each contour plot, the approximate location along the transect is indicated. The abbreviations stand for Hornsundb. – Hornsundbanken, Isfjordenb. – Isfjordenbanken, and PKF – Prins Karls Forland. The plot was generated using Ocean Data View Version 4.5.7 (https://odv.awi.de) gridding weighted averages.

We report the methane data collected during the three expeditions to the area (HE387 in August/September 2012, HE449 and HE450 in August/September 2015) as deviations from its atmospheric equilibrium concentration, which ranged from 2.6 to 3.5 nM depending on the temperature and salinity of the water sample^29^. The methane concentration anomalies of our 734 measurements ranged from −2.7 (below saturation) to 878.3 nM near seepage sites (Fig. 3), with an average concentration of 28.6 nM and a median of 9.5 nM.

At all stations (n = 68) except one, the elevated methane concentrations point to seafloor emissions. Methane measurements revealed a distinct plume situated near the seafloor at 30 locations, and 35 of the vertical profiles showed discrete maxima indicative of a release of methane from bubbles or of a horizon where methane from different sources convene. From the three PKF stations located farthest (~45 km) offshore, two profiles showed a single peak in the water column and one had no methane anomaly.

We divided our data into three shore-parallel transects located at increasing distances from the shore. Along our seaward transect (~40 km offshore, Fig. 3), methane concentrations were generally low, surface methane concentrations were mostly in equilibrium with the atmosphere and methane concentrations increased towards the seafloor reaching values of ~20 nM at Hornsundbanken and Isfjordenbanken. Along the middle transect (~15–40 km offshore, Fig. 3), highest methane concentrations were found associated with locations of intense seepage at 90 m water depth on Hornsundbanken, and at 80 m and 350 m water depth off PKF. The high methane concentrations on the middle transect decreased towards the coast (<15 km offshore, coastal transect in Fig. 3) with values comparable to those observed in the seaward transect. Only at PKF did methane concentrations reach values >100 nM. Surface methane concentrations were higher than the atmospheric equilibrium concentrations along the middle and coastal transect.

We linearly interpolated methane concentration anomalies obtained during the three cruises on a grid defined by the locations of the hydrocast-stations. The resulting grid extends over 320 km in the west-east direction (from easternmost to westernmost hydrocast-station), 630 km in the north-south direction (from southernmost to northernmost hydrocast-station), and encompasses up to 900 m of water depth (Supplementary Fig. S4). Slicing the grid in 10 m water layers, the highest inventories are clearly located at water depths from 100 to 300 m (Supplementary Fig. S5). The volume of the grid corresponds to 1.8 × 10^5^ km^3^ of which 19 × 10^3^ km^3^ contain methane, resulting in a total mass of 8.4 Gg.

### Stable carbon isotopic composition of methane

**Figure 4:**
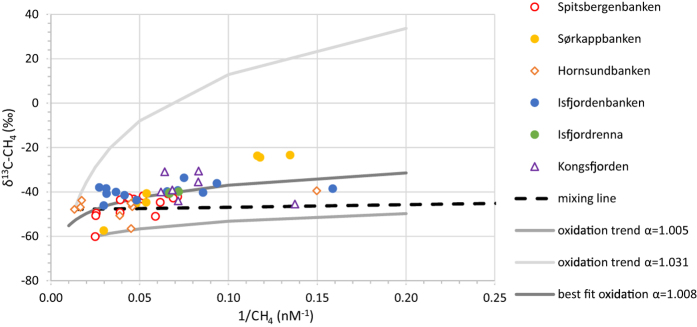
δ^13^C vs. 1/CH_4_ concentration for water samples. Samples are grouped according to their location at the Svalbard shelf. The stippled black line marks a mixing line between the sample with the highest methane concentration and background methane values of the ocean. The gray lines indicate microbial oxidation trends. The best fit to all data has an R^2^ of 0.36 with a δ^13^C-value of the source methane of −64‰.

Methane was extracted from water samples collected 1 to 11 m above the seafloor for stable carbon isotopic analyses. The δ^13^C-CH_4_ values of these samples range from −60.1‰ to −23.4‰ (Fig. 4). Methane relatively depleted in ^13^C (<−50‰) was found in a few gas samples collected from the southern part of the Svalbard shelf, at Spitsbergenbanken, Sørkappbanken, and Hornsundbanken, but most of the data show ^13^C-enrichments resulting in δ^13^C-CH_4_ values > −50‰. Samples with the highest methane concentrations collected at Hornsundbanken, Spitsbergenbanken, and Isfjordenbanken have δ^13^C-CH_4_ values of −46.2‰ (average of n = 3), −53.4‰ (n = 3), and −42.0‰ (n = 2), respectively.

### Methane oxidation rates

**Figure 5:**
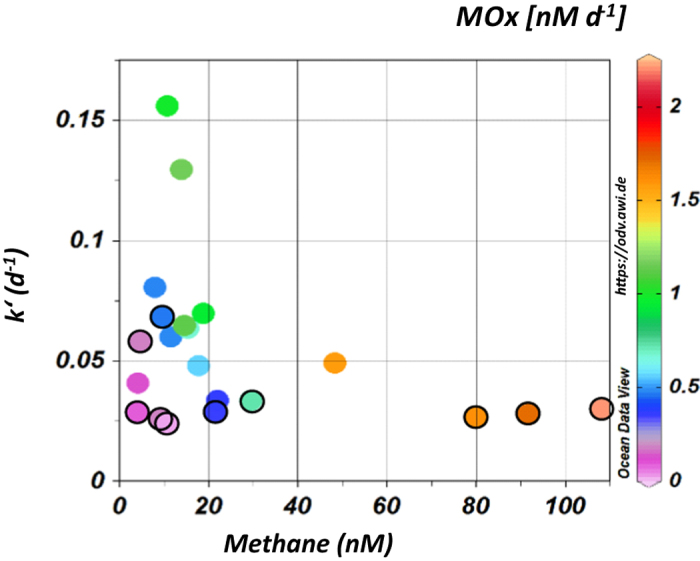
The relative activity of methane oxidizing microorganisms (k′) versus methane concentrations at Hornsundbanken (encircled) and Isfjordenbanken (without outline). The color code shows the resulting methane oxidation rates (MOx). Highest MOx-rates correlate with sites of gas emissions, i.e. highest methane concentrations. In addition, k′ and MOx increase down-current from Hornsundbanken to Isfjordenbanken. The plot was generated using Ocean Data View Version 4.5.7 (https://odv.awi.de).

We collected discrete water samples throughout the water column along two transects crossing Hornsundbanken and Isfjordenbanken (Fig. 5 and Supplementary Fig. S6) for quantification of the aerobic microbial methane oxidation (MOx). Highest MOx-rates were found near bubble emission sites, where they ranged between 1.6 and 2.2 nM d^−1^. These values are ~3 times higher than those measured in samples collected away from bubble emission sites. The elevated rates reflect the high methane concentrations near bubble emission sites; however, there is a noteworthy trend of increasing MOx-rates down-current (Fig. 5 and Supplementary Fig. S6), which is associated with an increase in relative methane oxidizing activity (k′). For example, MOx-rates across Hornsundbanken averaged 0.26 nM d^−1^, whereas at ~100 km down-current, across Isfjordenbanken, MOx-rates averaged 0.68 nM d^−1^. This increase reflects an enhanced relative activity (k′) from an average of 0.035 d^−1^ to 0.072 d^−1^.

### Sea-air flux of dissolved methane

**Figure 6:**
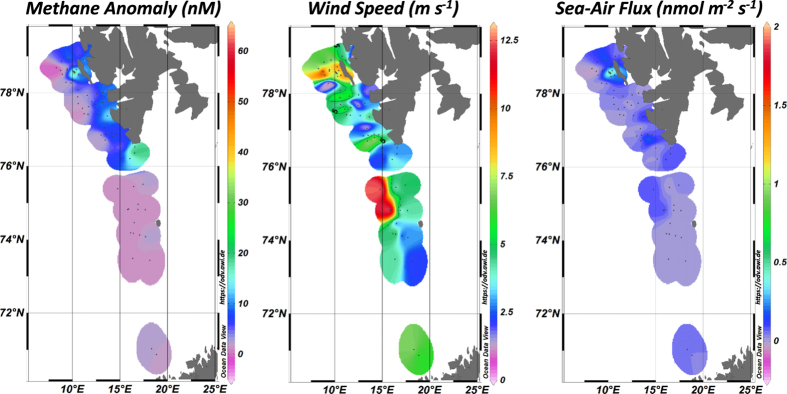
Surface methane anomalies, wind speed at 10 m above sea-surface during the time when water samples were collected, and sea-air flux of methane. The resulting sea-air flux of methane was elevated at the seepage sites off PKF, Hornsundbanken, and Sørkappbanken as well as in areas of wind speeds >5 m s^−1^. The plot was generated using Ocean Data View Version 4.5.7 (https://odv.awi.de) gridding weighted averages.

Sea-air fluxes were estimated for water sampled 3 to 11 m below the sea-surface, in the surface mixed layer. The estimated fluxes are strongly affected by wind speed and range between −0.2 and 2.0 nmol m^−2^ s^−1^ (Fig. 6), with a median of 0.021 nmol m^−2^ s^−1^. The highest fluxes were calculated for the shallow seepage area off PKF, Hornsundbanken, and Sørkappbanken. They are associated with methane concentrations >5 nM and with wind speeds >5 m s^−1^. Wind speed during our surveys ranged between 0.3 and 12.6 m s^−1^, however, 59% of the sampling was done at low wind speeds (<5 m s^−1^). Consequently, 73% of all estimated methane fluxes to the atmosphere range between 0 and 0.1 nmol m^−2^ s^−1^.

## Discussion

Our results greatly extend knowledge of the areal coverage of methane seepage west of Svalbard. To date, methane gas emissions have only been documented at Vestnesa Ridge and offshore PKF, both areas are located on the Svalbard continental margin. At the 1200 m deep Vestnesa Ridge, flares were found above pockmarks^30^. Offshore PKF, gas emissions were reported at the upper slope (396 m), at the outer shelf (240 m), and at the Forlandet moraine complex (80–90 m)^14^,^25^. In addition, in the SW Barents Sea, gas flares were mapped along a segment of the Ringvassøy Loppa Fault Complex near the Snøhvit and Albatross gas field^31^. Bubble release was also documented at the Haakon Mosby Mud Volcano, located in 1270 m water depth at the center of the Bjørnøya slide scar on the SW Barents Sea slope^32^. Here we report on additional point sources of methane over a distance of 630 km, from the latitude of Bjørnøya to Kongsfjordenna. Seep clusters were found west off Bjørnøya, at Sørkappbanken, and at Hornsundbanken, but we also discovered several single seeps between these clusters (Fig. 1a). Since this extensive seepage region covers the Svalbard shelf, we designate it as the Svalbard plume.

Methane rich fluids feeding the Svalbard plume appear to migrate either along faults, along stratigraphic boundaries or through a combination of these two structures. Most of the flares mapped in this study are located in the vicinity of the HFZ; a few single flares were found in Kongsfjordrenna, near the Knølegga Fault Zone, and along the northern edge of the Kveithola Trough. Knies *et al*.^33^ and Damm *et al*.^34^ had previously noted a relationship between high methane concentrations and the HFZ at the western Spitsbergen shelf. In contrast, Rajan *et al*.^35^ suggested that, since the fluids expelled at ~250 m water depth offshore PKF align with the outcrop of a glacigenic sequence, fluid migration is likely occurring along dipping strata in the prograding sequence. Since the Barents Sea Ice Sheet extended to the slope edge from northern Norway to northern Svalbard^36^, glacigenic stratigraphy could provide a pathway for ascending fluids not only at the PKF, but also farther south at Hornsundbanken, Sørkappbanken, and Spitsbergenbanken. Our surveys could not identify fluid migration along stratigraphic boundaries; but our data indicate that the majority of the gas emissions follow the HFZ.

The majority of the gas emission sites feeding the Svalbard plume are located on bathymetric highs with only a few single flares found within the troughs. We speculate that the fine-grained postglacial deposits in the troughs seal structural pathways or at least limit methane-rich fluid migration to the seafloor. In the Storfjordrenna and the Kveithola Trough, glacigenic diamicton is overlain by meter-thick sediments originating from melt-water-pulses and contour currents^37^. Isfjordrenna and Bellsund contain stiff diamicton beneath Holocene and glaciomarine sediments, respectively^24^. In contrast, bathymetric highs or banks expose only the matrix-supported diamicton, which is imaged in the sub-bottom profiles as having none or chaotic reflectors and is interpreted as subglacial till partially reworked by winnowing and bioturbation^24^. Although seepage appears associated with the over-consolidated, dense diamicton, it is very likely that this material does not allow fluid migration through the rock itself. Hence, we assume fluid migration along structural pathways, created by faulting along the HFZ.

A notable exception to the seepage typically observed in topographic highs is Isfjordenbanken, where there are fewer flares emanating from the seafloor than from the other banks. Multi-channel seismic data document the presence of a sediment filled, N-S striking graben (the Bellsund Graben) beneath the uppermost layer of Isfjordenbanken^20^. It is possible that the infill of the Bellsund Graben blocks or reduces fluid migration pathways that are open at the other bank-areas that lack the additional sediment deposits.

The observed bubble emissions generate the Svalbard plume (Fig. 3). Based on analyses of the relative position of each CTD-station and flare observation, we can estimate that 80% of the methane profiles are located ~30 km away from the nearest flare site. Methane plumes of ~30 km lengths have previously been observed down-current from the Coal Oil Point (COP) seep field^38^ and from the Deepwater Horizon well^39^.

Furthermore, our observed increase of methane concentrations towards the seafloor, methane maxima a few meters above the seafloor, and sudden concentration peaks in the vertical profiles indicate a gaseous methane input from the seabed. About half of the 68 methane profiles that sampled the entire water column showed an increase in methane concentrations towards the seafloor, where most of the methane dissolves from bubbles^8^. At 17 stations methane maxima were observed a few meters above the seafloor, which suggest gas deposition from bubble dissolution. As documented by Leifer *et al*.^40^, when gas bubbles rise through the water column they expand due to a decrease in hydrostatic pressure and at the same time decrease in volume due to dissolution. Because of the dominant decrease in bubble size, the net buoyancy force decreases during ascent and methane is deposited at water depths where the hydrostatic equilibrium is reached and bubbles cannot continue moving upward. Discrete peaks in our methane profiles may originate from transport and dissolution of gas bubbles, either by natural gas deposition at depth from bubble dissolution, lateral or episodic bubble transport, or by bubble dissolution within the Niskin bottle during CTD upcasts^41^.

Methane sources in the water column associated with the oxygen minimum zone or a phytoplankton bloom were not apparent. An oxygen minimum zone, defined as water with <20 μM O_2_ (transition of O_2_ to NO_3_^−^-respiration)^42^,^43^, was not observed offshore Svalbard, as the lowest oxygen concentration measured was 270 μM. A fluorescence peak in the uppermost 50 m of the water column was recorded in our CTD casts, but there was no correlation between the high methane concentrations and the chlorophyll a maxima. Therefore, we conclude that the phytoplankton blooms here are not a significant methane source.

The total mass of the methane plume generated by bubble emissions was estimated to be 8.4 Gg methane. Compared to the 200 Gg of methane injected during the Deepwater Horizon Accident^44^, the Svalbard plume mass appears insignificant. However, the estimated mass of methane carried by the Svalbard plume is larger than the down-current methane plume that originates from the Coal Oil Point seep field (~50 Mg)^38^, methane inventories of Hydrate Ridge (37 Mg)^41^, and much larger than the methane release from mud diapirs offshore Costa Rica (0.2–8 kg)^45^. Thus, the Svalbard plume is on the upper end of the known range of dissolved methane in plumes originating from natural seepages.

The two main sinks of dissolved methane in the oxygenated water column are aerobic microbial methane oxidation and sea-air exchange. Using our MOx-rate measurements and estimates of the sea-air flux, we calculated the fractions of methane in the plume consumed within or transferred out of the ocean. Our MOx-rates range between 0.01–2.19 nM d^−1^, which include the spatiotemporal variability observed between Hondsundbanken and Isfjordenbanken. This range also accounts for the variability observed between the Arctic Surface Water and the Atlantic Water, as Arctic Surface Water hosts a greater abundance of methanotrophic bacteria^46^. Our MOx-rates are consistent with previously reported rates of 0.01–1.95 nM d^−1^ in Storfjorden^47^ and up to 3.2 nM d^−1^ in the PKF region^46^. By multiplying our MOx-rates by the water volume comprising the plume, we estimate the fraction of methane that is oxidized in the plume to be 0.02–7.7% per day (median 1.8% per day).

To quantify the methane lost to the atmosphere, we use our sea-air fluxes of −0.13 to 1.79 nmol m^−2^ s^−1^. This range includes the spatiotemporal variability of methane concentrations (−1.8 to 62.3 nM) and wind speed (0.3–12.6 m s^−1^) observed over the course of three cruises. The surface methane concentrations were measured mainly in Melt/Surface Water, hence, our data do not include higher or lower sea-air fluxes resulting from a prevalence of Atlantic Water or Arctic Surface Water, as suggested by Steinle *et al*.^46^. If we extrapolate over the surveyed area we arrive at values of −0.04 Gg d^−1^ to 0.5 Gg d^−1^, which translate to a potential daily maximum of 5.9% of the methane in the plume being transferred to the atmosphere. However, during our summer cruises wind speed were <5 m s^−1^, which correspond to a mean of the sea-air fluxes of 0.02 nmol m^−2^ s^−1^. If we use this mean value, which corresponds to 59% of the survey time, we get a value for methane fraction of 0.07% transferred to the atmosphere per day. Therefore, during the summer a larger fraction of the methane appears to be microbially consumed and a smaller fraction leaves the ocean via sea-air transfer, especially during low wind speed periods.

Our estimates on the overall methane partitioning along the continental shelf match studies by Graves *et al*.^48^, who concentrated on the area offshore PKF, close to the landward limit of the gas hydrate stability zone. They showed that 60% of the methane inventories are consumed before methane is mixed in the surface mixed layer and can be transferred to the atmosphere. Their sea-air fluxes range between 0.046 and 0.23 nmol m^−2^ s^−1^, a range that is similar to estimates by Lund Myhre *et al*.^49^, who reported a median ocean-atmosphere methane flux of 0.04 nmol m^−2^ s^−1^ for the area offshore PKF. Our estimates of sea-air fluxes from the PKF area (−0.13 to 1.79 nmol m^−2^ s^−1^) and the Hornsund seep area (0–0.28 nmol m^−2^ s^−1^) are of similar magnitude, while our estimated flux over the entire Svalbard margin is lower than the one from the area offshore PKF. An increase in wind speed during winter will deepen the surface mixed layer and might considerably increase the flux to the atmosphere, as was shown for a seep field in the central North Sea^50^. Furthermore, it should be noted, that 70% of our observed bubble emission sites occur at water depth of <120 m, and such shallow depths facilitate a direct release of methane to the atmosphere from ascending bubbles. Gas transfer is not included in our estimates, which are based on dissolved methane concentrations. Taking our low estimate of 0.02 nmol m^−2^ s^−1^, assuming a constant methane inventory, and neglecting any temporal variability of gas emissions, microbial methane oxidation, and wind pattern, we arrive at a minimum value of 18% of the methane plume that would be transferred to the atmosphere per year, which translates to a flux in the order of 1.5 Gg yr^−1^.

To constrain the methane sources feeding the Svalbard plume, we measured the stable carbon isotopic composition of methane dissolved in water samples collected 1–11 m above the seafloor. Methane concentrations in these samples were above atmospheric equilibrium, and their δ^13^C-CH_4_-values range from −60.1‰ to −23.4‰. These stable carbon isotopic ratios might reflect changes due to mixing with background methane transported by ocean currents and/or they may arise from aerobic microbial oxidation either in the water column and/or surface sediments. If mixing was responsible for the observed stable carbon isotopic composition, then our data would plot along a straight line that connects highest methane concentrations and their δ^13^C values with methane background values (<3 nM) in a δ^13^C versus 1/CH_4_ plot^51^ (Fig. 4). Background values of δ^13^C range between −44.5 to −44.9‰ in the surface ocean <200 m^52^,^53^ and increase due to ongoing methane oxidation as water mass ages to values of −32 to −41‰ below the surface mixed layer^52^. Our data do not align with a mixing line, and thus indicate that in the Svalbard plume mixing with background methane does not significantly alter the stable carbon isotopic composition of the methane source.

We note that samples that contain higher concentrations of methane have more negative δ^13^C value, i.e. are more enriched in ^12^C, than those with lower methane concentration (Fig. 4). The enrichment in ^13^C in these samples with low methane concentration might reflect consumption of methane by microorganisms, which preferentially take up ^12^C-CH_4_ leaving ^13^C-enriched methane behind. As a first approximation, we assume that the methane in all areas investigated in this study originates from the same carbon source; an assumption solely based on the observation of a similar geological setting. We calculated a best fit for an oxidation trend using the Rayleigh fractionation after Coleman *et al*.^54^, fractionation factors (α) between 1.005 and 1.031^55^, a methane concentration range between 60–790 nM, and δ^13^C-values from −40 to −80‰. The best fit had an R^2^ of 0.36 with α equal to 1.008, a concentration of the source methane of 316 nM, and a δ^13^C-value of the source methane of −64‰. As a second step, we derived oxidative trends for separate source regions such as Spitsbergenbanken including Kveithola Trough (R^2^ = 0.49, α = 1.01, [CH_4_]-source = 207 nM, δ^13^C-source = −69‰) and Sørkappbanken (R^2^ = 0.98, α = 1.02, [CH_4_]-source = 95 nM, δ^13^C-source = −80‰). However, we could not obtain an oxidative trend based on the data of Hornsundbanken, Isfjordenbanken, Isfjordrenna, and Kongsfjordrenna. Based on these relationships we conclude that in the southern areas >65% of the methane originally discharged at the seafloor was oxidized before we sampled the water column (81–93% for the Spitsbergenbanken fit and 65–92% for the Sørkappbanken fit).

The derived δ^13^C-sources with an estimated stable carbon isotopic composition of −64 to −80‰, coupled with a lack of detection of any higher hydrocarbons, point to microbial generated methane^56^ for the Svalbard plume. However, we do not discount the possibility that higher hydrocarbons could have been removed by migration^57^ or preferential microbial oxidation^58^. Oxidative trends could only be fitted to part of the data, therefore in the northern regions there might be less overprinting by microbial oxidation than we estimated for the southern regions. We cannot rule out the possibility that a fraction of the methane is thermocatalytically generated, nor the possibility that upward migration of methane induced isotopic fractionation^57^ resulting in more or less ^12^C enriched methane depending on the fluid pathway. Answers to these outstanding questions necessitate data from deep sediment cores.

Although we cannot fully establish the methane source feeding the Svalbard plume, our δ^13^C-methane data are consistent with previous measurements from various sites west of Svalbard. A mixture of thermocatalytic and microbial methane was postulated by Knies *et al*.^33^ as the source of adsorbed methane in shelf sediments off Spitsbergen, whereas they argued that the free gas composition was likely altered by microbial processes. Sahling *et al*.^25^ documented that methane of seepage sites >230 m off PKF has δ^13^C-methane values of −55.7‰, suggesting a microbial origin, whereas the methane of a seepage site at 80–90 m water depth was more ^13^C-enriched (−43.5‰). Gas hydrates collected at the slope (890 m, −54.6‰)^59^ and water column samples from the 230 m seepage site on the shelf also point to a larger microbial contribution at the deeper sites^60^. Damm *et al*.^34^ reported on ^13^C-enriched methane discharging from seeps on top of sandy and gravelly banks and ^12^C-enriched methane escaping from small troughs filled with silty and clayey sediments.

## Conclusions

Water column investigations along the Svalbard continental margin from Bjørnøya to Kongsfjorden, revealed gas emission clusters and single seep sites that generate a methane plume seemingly hundreds of kilometers long. This Svalbard plume is similar in magnitude to the COP plume in the Santa Barbara Basin. The methane release follows the trace of the Hornsund Fracture Zone, and the correspondence of flare locations with hard-sediment reflections in bathymetric highs point to transport along structural fracture zones and to a prevalence of the methane discharge in locations not covered with significant sediment accumulations. A fraction of the 8.4 Gg of dissolved methane in the Svalbard plume was oxidized in the water column and an even smaller fraction was transferred to the atmosphere during the low wind speed conditions during summer.

Large methane plumes such as the Svalbard plume and the COP plume appear to be natural phenomena. The gas emissions generating these plumes are located mainly above the gas hydrate stability zone, therefore, these plumes appear not directly linked to gas hydrate dissociation. Our results point to a geologically controlled natural release of methane along the Svalbard margin, which does not appear to be linked to an anthropogenic forcing. Furthermore, our results indicate that most of the greenhouse gas remains in the ocean during summer time, where part of the dissolved gas is oxidized by microbes. However, anthropogenic forcing can influence methane release manifold and their single and combined effects remain speculative. On the one side, sea level rise would increase hydrostatic pressure and thus decrease gas emissions, and longer seasonal stratification would prolongate the time methane can be oxidized in the ocean. On the other side, elevated melting of glaciers and thus intensified organic matter transport from land, and possible higher phytoplankton production rates due to global warming and/or ocean acidification would increase methane production. Thus, the need to further qualify and quantify the source term for the methane peaks observed in ice cores remains an important task for modern geoscientists.

## Methods

All data used in this study were collected during three RV *Heincke* expeditions. The first cruise (HE387) was conducted in August/September 2012 and investigated the methane seepage area offshore PKF; the second and third cruises (HE449 and HE450) took place in August/September 2015, and extended the survey area. The flare distributions obtained during HE387 cruise off PKF and included in this study were already published in Sahling *et al*.^25^. During HE449 the shelf and shelf edge were explored from Sørkapp in the south to Isfjorden in the north and during the HE450 our survey extended from northern Norway in the south to Kongsfjorden in the north (see cruise tracks shown in Supplementary Fig. S1).

### Hydroacoustic Profiling

Sub-seafloor structures were imaged with the sediment echosounder *Innomar* SES-2000 medium permanently installed on the hull of the RV *Heincke*. The system operates at a primary frequency of 100 kHz and we chose a secondary frequency of 6 or 8 kHz. The *Innomar* echosounder was run continuously during this cruise; the data were stored in *.ses and *.raw-formatted files and replayed with the program ISE 2.9.2 post-processing software (provided by *Innomar*).

Active gas emissions, which become visible as flares in water column echograms, were recorded with both a split beam echosounder *Simrad* EK60 with 38 kHz frequency and a *Kongsberg* EM710 multibeam echosounder operating at a frequency of 70–100 kHz. For the precise localization of individual flares, the water column data were post-processed using the Fledermaus tools FMMidwater, DMagic, and the 3D Editor (©QPS). Seafloor origins of individual flares were identified as points of highest amplitudes near the seafloor. The coordinates of these points were extracted using the FMGeopicker and subsequently plotted on top of the bathymetry using ArcGIS 10.2 (©ESRI). For visualization of flare deflections and bubble rising heights, selected flares were extracted from the water column records of the multibeam echosounder as point data, and were edited using the 3D Editor of Fledermaus.

### Water column sampling

Seawater was sampled at 68 stations to quantify the dissolved methane distribution along the shelf and shelf edge off Svalbard and the Barents Sea (Fig. 3). Seawater was sampled using a rosette equipped with 12 × 5 l Niskin bottles mounted on a frame that holds a Sea-Bird SBE 911 plus and a SBE 43 oxygen sensor for online monitoring of salinity, temperature, pressure, and dissolved oxygen. The accuracy of the conductivity, temperature, and pressure was ± 0.0003 S/m, ±0.001 °C, and ±0.015%, respectively. Winkler titration of dissolved oxygen at three locations validated the sensor data. The mean difference of all the samples was 0.03 ml l^−1^.

### Methane concentrations

Twelve water depths were sampled at each station for methane concentration analysis. For determination of methane concentrations during HE387, gas was extracted from sampled water using the vacuum extraction method after Lammers and Suess^61^. Briefly, 700 to 750 mL of seawater were directly collected from Niskin bottles into pre-evacuated (1 × 10^−3^ mbar) 1 l-glass bottles (Schott DURAN) with a gas tight closure. By injection of degassed brine, the gas was transferred into a burette where the gas volume can be quantified. The extracted gas was further transferred into 20 ml serum glass vials pre-filled with saturated NaCl solution and stored at 4 °C. Analysis of methane concentrations in the extracted gas was conducted onboard with a 6890N gas chromatograph (Agilent Technologies) equipped with a capillary column and connected to a Flame Ionization Detector, as described in Pape *et al*.^62^. Calibrations and performance checks of the analytical system were conducted regularly using commercial pure methane standards. The coefficient of variation determined for the analytical procedure was less than 2%.

During HE449 and HE450 methane concentration measurements were performed using the Greenhouse Gas Analyzer ‘Enhanced Performance’ with ‘Syringe Injection Mode’ manufactured by Los Gatos Research (LGR), California, using the procedure described in detail by Geprägs^63^. The instrument uses conventional laser absorption spectroscopy, where the absorption of the infrared laser beam directed through the sample is used to calculate the mole fraction of methane in the gas. We used three stock standards (1, 10 and 100 ppm methane) and dilutions of these standards to calibrate the instrument in the range from 0.07 to 100 ppm. Water samples were collected by filling two 140 ml syringes, each outfitted with a valve, directly from the Niskin bottles. The syringes were flushed and filled with 100 ml of seawater without any air bubbles. The syringes were left to equilibrate to room temperature or the temperature of the sampled water was measured to avoid a long equilibration time in the case of low temperatures (<5 °C). A headspace was generated in each syringe by drawing 40 ml of Zero Air into the syringe. Syringes were shaken vigorously for over 1.5 minutes to allow for equilibration between water and headspace^64^. The combined gas volume of 80 ml was injected in the GGA followed immediately by 60 ml injection of Zero Air, which is needed to reach the required volume of 140 ml in the instrument chamber of the GGA.

The GGA calculates methane concentrations based on its internal calibration. To minimize potential errors caused by shifts in the internal calibration of the instrument we ran calibration curves for each batch of samples analyzed. The dissolved methane concentration in the samples was calculated according to the headspace formulation detailed in Magen *et al*.^64^ and the required Bunsen coefficient was calculated based on the salinity and temperature of the sample. The reproducibility of the method is <2.5%. In comparison with the vacuum extraction method, the GGA yielded 18% higher values, which might be due to the extraction efficiency of 90 ± 6%^65^.

### Stable carbon isotope composition of methane

From water, collected 1–11 m above seafloor using the CTD-rosette, gas was extracted and analyzed for the stable isotope composition of methane. Gas was extracted from sampled water using the vacuum extraction method briefly described above for methane concentration analysis during HE387. The stable carbon isotope analysis was performed with an isotope ratio mass spectrometer (IRMS; Finnigan Delta XP plus) at the Alfred Wegener Institute, Bremerhaven, Germany. To concentrate the methane for analysis, the gas was purged and trapped with PreCon equipment (Finnigan). All isotopic ratios are presented in the δ notation against the Vienna Pee Dee Belemnite (VPDB) standard and have an analytical error of <1‰. Replicates have an error of ±1‰.

### Methane oxidation rates

Methane oxidation (MOx) rates were determined during HE449 from *ex situ* incubations of water samples in 120 ml serum vials. Sample collection and incubation were performed as described in Bussmann *et al*.^66^. Briefly, triplicate samples were collected and 100 μl of ^3^H-labeled methane (~200 kBq) in N_2_ were added to each sample. After shaking the bottles to equilibrate the tracer with the water, the samples were incubated in the dark for 48 h at 2.5 ± 1.4 °C. Incubation was stopped by adding 0.3 ml of 25% H_2_SO_4_. Then the total activity (^3^H-CH_4_ + ^3^H-H_2_O) in an 3 ml aliquot was measured by liquid scintillation counting; the activity of ^3^H-H_2_O was measured after sparging the sample for 30 min with air to remove excess ^3^H-CH_4_, so that the net amount of ^3^H-CH_4_ consumption can be estimated. Counting was performed on board with a Hidex scintillation counter and Ultima Gold™ LLT scintillation cocktail. Coefficient of variation was on average 26% for replicate samples.

MOx rates were calculated assuming first-order kinetics:


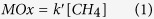


where *k′* is the effective first-order rate constant calculated as the fraction of labeled methane oxidized per unit time, and [CH_4_] is the *in situ* methane concentration. To verify first order kinetics we conducted time series incubations and measured the tracer consumption after 24, 47 and 61 h. In addition, control samples were frequently taken and poisoned immediately after the addition of the tracer. The limit of detection (LOD) calculated as described in Bussmann *et al*.^66^ was 0.025 nM day^−1^.

### Inventory and sea-air flux estimates

In order to evaluate the extent of the methane plume and its impact to the atmosphere, we calculated the inventory and the sea-air flux of methane. For derivation of the inventory, the discrete water sample data set were interpolated by Matlab™ via the TriScatteredInterp function using linear interpolation into a grid of 10 km x 10 km x 10 m. The interpolated data was used to calculate the methane inventory (*I*):


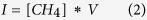


where [CH_4_] is the interpolated value for a 10^9^ m^3^ grid cell volume (*V* = 10 km × 10 km × 10 m).

The sea-air flux (*SAF*) was calculated as:


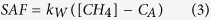


where *k*_*W*_ is the gas transfer velocity in cm h^−1^, [CH_4_] is the measured concentration of methane and *C*_*A*_ is the methane concentration in atmospheric equilibrium, both in nM. We calculated *k*_*W*_, which depends on wind speed and the temperature-dependent Schmidt number of the gas, using parameterization developed by Wanninkhof 67, Nightingale *et al*.^68^, McGillis *et al*.^69^, Ho *et al*.^70^, and Wanninkhof *et al*.^71^. The error associated with the different *k*_*W*_ estimates yield an average flux uncertainty of 17%, which increases at wind speeds <3 m s^−1^ to 116%. Wind speed was recorded 22 m above sea level onboard with a precision of 20% and corrected to the standard height of 10 m by applying the power law:


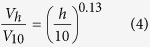


where *V*_*h*_ is the mean wind speed at a height of *h, V*_*10*_ is the mean wind speed at 10 m, and *h* is the effective height of the anemometer above mean sea level (http://www.metoffice.gov.uk/media/pdf/s/p/OH_Chapter51.pdf). *C*_*A*_ was derived using the mean atmospheric methane concentration of Ny-Alesund, Svalbard, ZEP, of August/September 2012 and 2015 (1.895 ppm, http://www.esrl.noaa.gov/gmd/dv/data/), the Bunsen solubilities are those given by Wiesenburg and Guinasso^29^ using measured ocean temperature and salinities. The sea air flux was calculated for all near surface water samples, which were collected in 3–11 m water depth below the sea-surface in the upper mixed layer. As the sea air flux depends strongly on wind speed, the crucial uncertainties of this flux are associated with wind speed measurements and the parameterizations of the gas transfer velocity, which yield an overall uncertainty of ~60%.

## Additional Information

**How to cite this article:** Mau, S. *et al*. Widespread methane seepage along the continental margin off Svalbard - from Bjørnøya to Kongsfjorden. *Sci. Rep.* **7**, 42997; doi: 10.1038/srep42997 (2017).

**Publisher's note:** Springer Nature remains neutral with regard to jurisdictional claims in published maps and institutional affiliations.

## Supplementary Material

Supplementary Material
