# Supplementary material for: Widespread methane seepage along the continental margin off Svalbard - from Bjørnøya to Kongsfjorden
Source: Sci Rep. 2017 Feb 23;7:42997. doi: 10.1038/srep42997 (PMC5322355; doi:10.1038/srep42997)
Supplement: Supplementary Material [file srep42997-s1.pdf]

Supplementary Material to:

Widespread methane seepage along the continental margin off Svalbard - from Bjørnøya to Kongsfjorden

S. Mau*[1], M. Römer[1], M. E. Torres[2], I. Bussmann[3], T. Pape[1], E. Damm[3], P. Geprägs[1], P. Wintersteller[1], C.-W. Hsu[1], M. Loher[1], and G. Bohrmann[1]

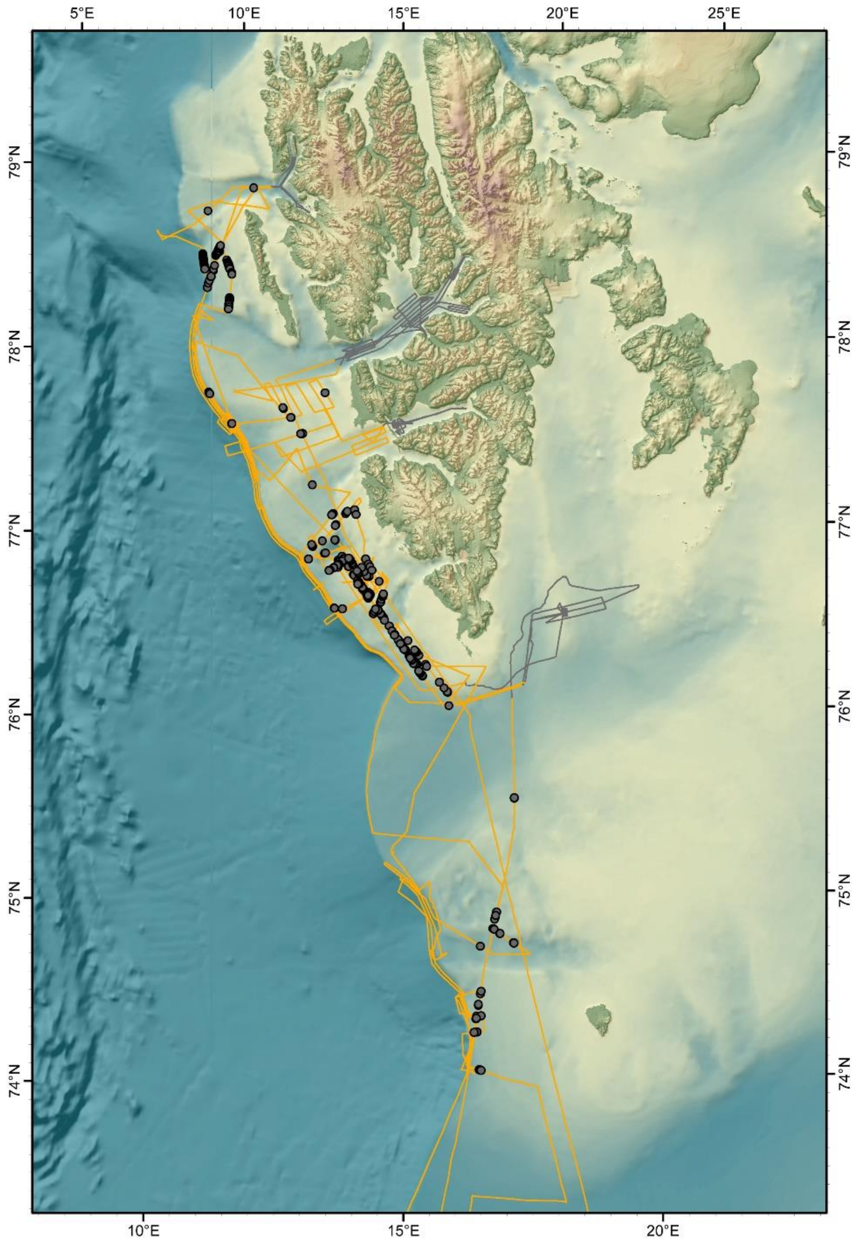

Supplementary Fig. S1: Flares and cruise tracks of HE449 and HE450. Gray dots mark flare locations as shown in Fig. 1 and yellow line shows cruise tracks of HE449 and HE450 on the shelf. Cruise tracks in the fjords are shown in gray and are not subject of this study. The map was generated using ArcGIS 10.2 (©ESRI).

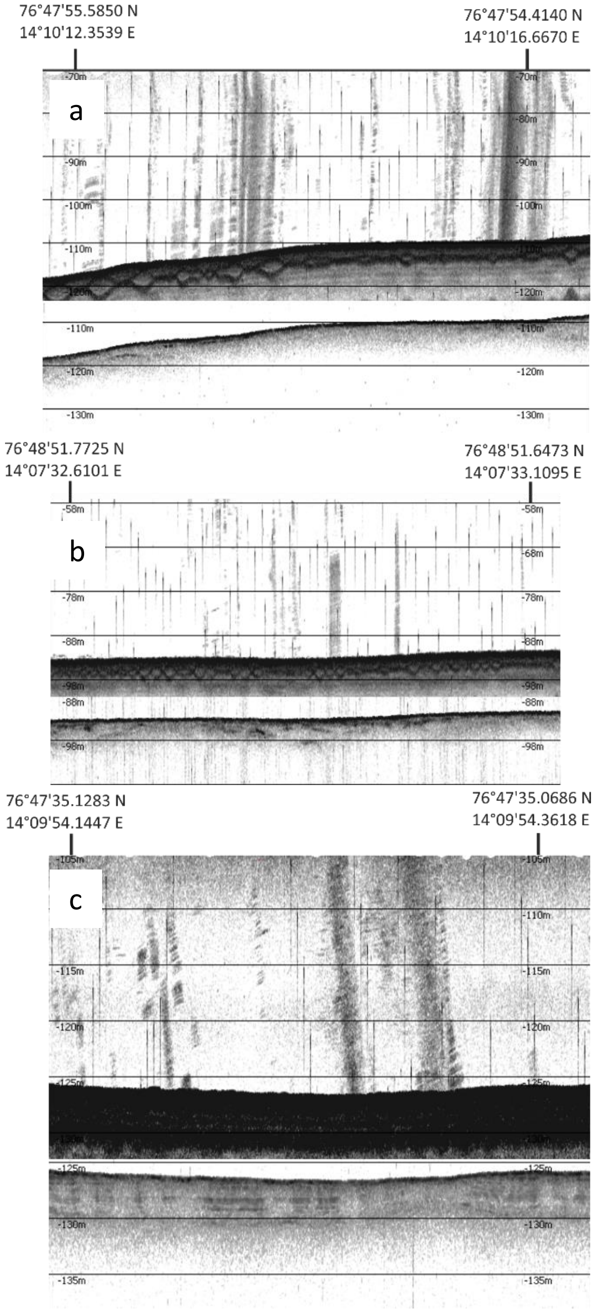

Supplementary Fig. S2: Examples of EK-60 water column profiles and SES-2000 sub-bottom profiles (upper and lower part of figures, respectively) showing flares above different grounds. The majority of the flares occurred (a) on top of hard ground without any seafloor penetration and (b) in areas of slight seafloor penetration with chaotic structure. Less flares were observed (c) in locations of slight to moderate seafloor penetration with parallel sub-seafloor reflectors.

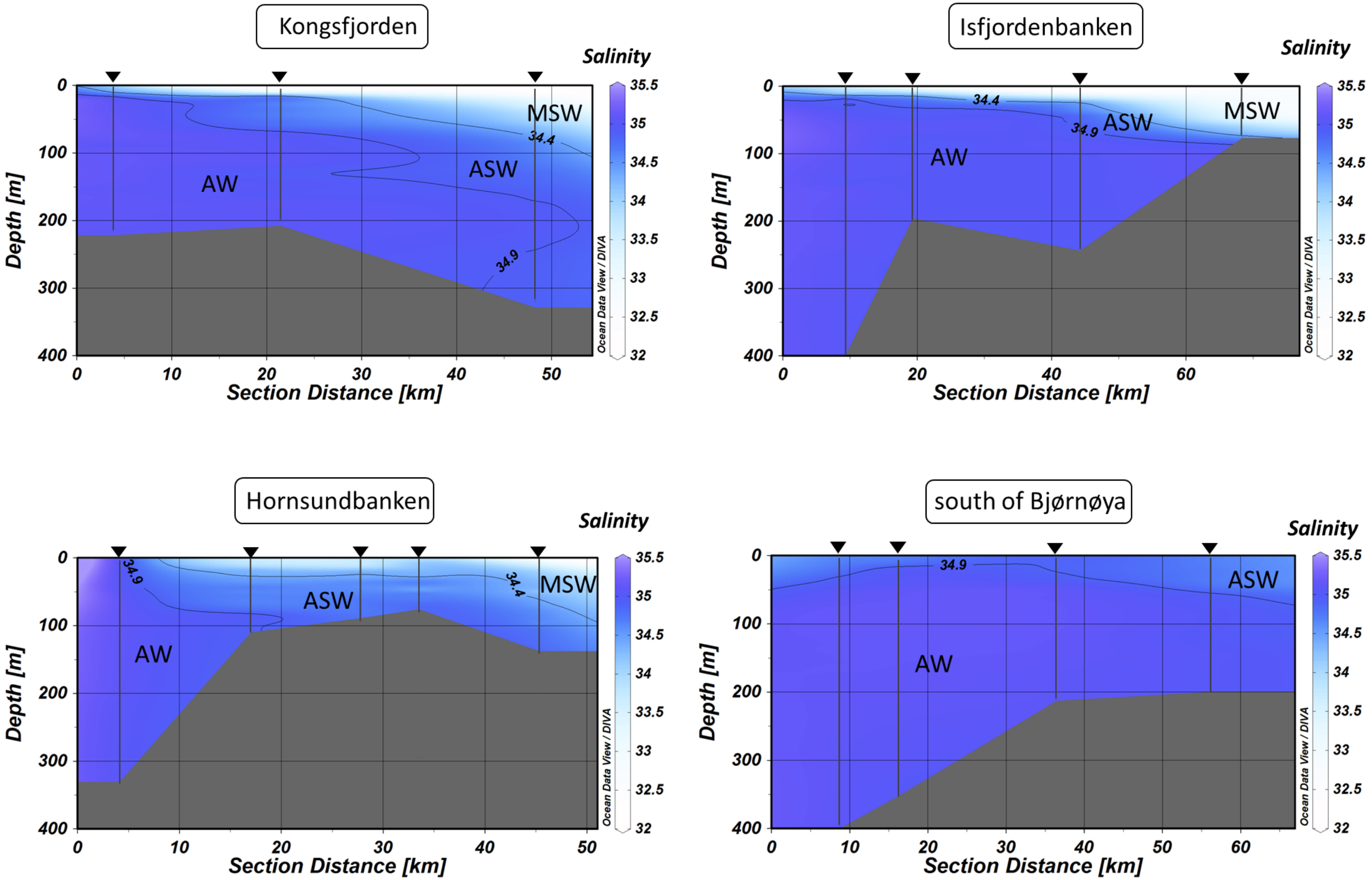

Supplementary Fig. S3: Salinity transects across the shelf. The locations of the transects are shown in Fig. 2. Water masses are abbreviated: AW – warm Atlantic Water, ASW – cold Arctic Surface Water, and MSW – Melt/Surface Water. The plot was generated using Ocean Data View Version 4.5.7 (https://odv.awi.de) and DIVA gridding.

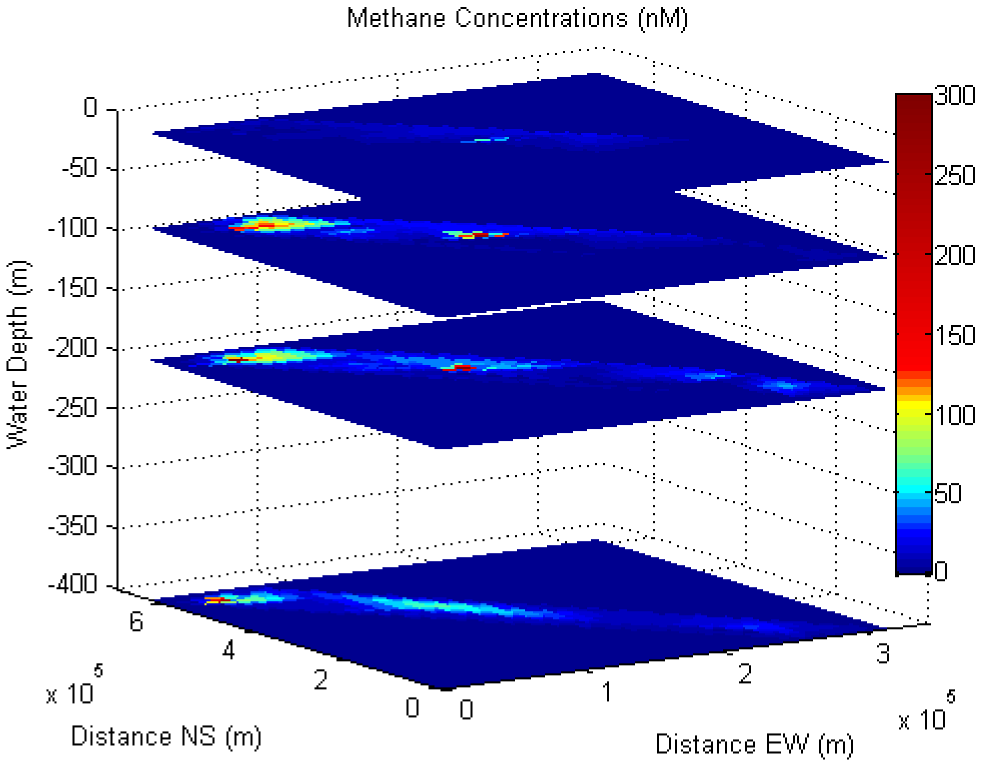

Supplementary Fig. S4: Gridded methane concentrations at 0 m, 90 m, 200 m, and 400 m water depth. Distinct are the two major seepage areas of Hornsundbanken and offshore PKF. Methane concentrations decrease rapidly towards the sea-surface.

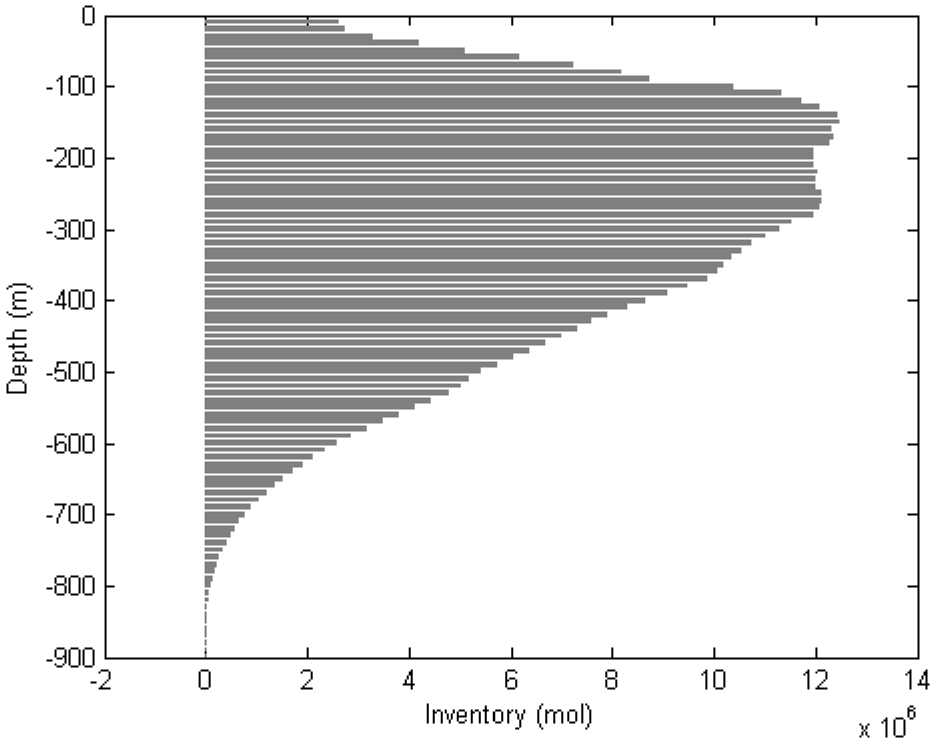

Supplementary Fig. S5: Methane inventories in 10^6 mol per 10 m water layers. Highest inventories were derived in 100-300 m water depth.

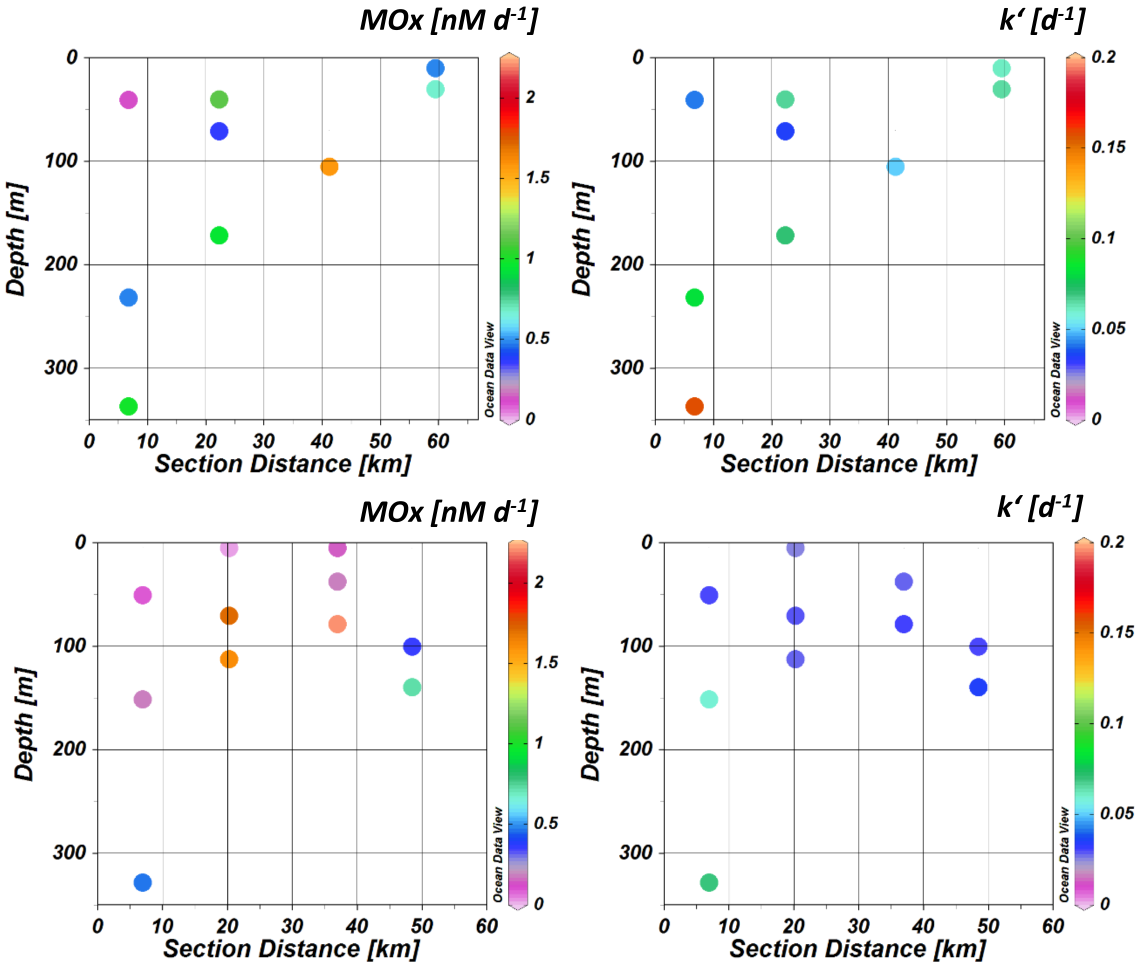

Supplementary Fig. S6: Methane oxidation rates (MOx) and the relative activity of methane oxidizing microorganisms (k') across Isfjordenbanken (upper graphs) and Hornsundbanken (lower graphs). Locations of the transects are shown in Fig. 2. Highest MOx were found near gas emission sites, i.e. where methane concentration are highest, however, there is also a general trend of increased k' and MOx down-current from Hornsundbanken to Isfjordenbanken. The plot was generated using Ocean Data View Version 4.5.7 (https://odv.awi.de).
